# Prognostic role of long non-coding RNA TUG1 expression in various cancers: a meta-analysis

**DOI:** 10.18632/oncotarget.20037

**Published:** 2017-08-08

**Authors:** Yongping Zhou, Yuxuan Lu, Runmin Li, Nana Yan, Xiding Li, Tu Dai

**Affiliations:** ^1^ Department of Hepatobiliary, Wuxi Second Hospital, Nanjing Medical University, Wuxi, China; ^2^ School of Medicine, Tongji University, Shanghai, China; ^3^ Department of Endocrinology, Hebei General Hospital, Shijiazhuang, China; ^4^ Department of General Surgery, Wuxi Second Hospital, Nanjing Medical University, Wuxi, China

**Keywords:** long non-coding RNA TUG1, cancer, prognostic

## Abstract

Several studies were conducted to explore the prognostic role of long non-coding RNA taurine upregulated gene 1 (lncRNA TUG1) expression in various cancers, with contradictory. This study aims to summarize the prognostic role of lncRNA TUG1 expression in various cancers. Embase, PubMed and Cochrane Library were completely retrieved. The cohort studies focusing on the prognostic role of lncRNA TUG1 expression in various cancers were eligible. The endpoints were overall survival (OS) and clinicopathological parameters. 9 studies involving a total of 1,078 patients were identified. The results showed that high lncRNA TUG1 expression was obviously associated with worse OS when compared to the low lncRNA TUG1 expression (HR = 1.37, 95% CI = 1.07–1.76, *P* = 0.01; I^2^ = 85%). However, No distinct relationship was observed between the lncRNA TUG1 expression and age (OR = 0.99, 95% CI = 0.76–1.28, *P* = 0.92; I2 = 4%), gender (OR = 0.92, 95% CI = 0.70–1.22, *P* = 0.57; I^2^ = 0%), diameter (OR = 0.83, 95% CI = 0.34–2.01, *P* = 0.67; I^2^ = 85%), smoking (OR = 1.09, 95% CI = 0.37–3.21, *P* = 0.87; I^2^ = 73%), TNM stage (OR = 0.60, 95% CI = 0.25–1.43, *P* = 0.25; I^2^ = 86%) and lymph node metastasis (OR = 1.07, 95% CI = 0.47–2.45, *P* = 0.87; I^2^ = 86%). In conclusion, it was revealed that high lncRNA TUG1 expression is an unfavorable predictor of OS in patients with cancers, and lncRNA TUG1 expression is a promising prognostic biomarker for various cancers.

## INTRODUCTION

Cancer has become a major public health problem heavily threatening human health and life [1, 2]. Early diagnosis and treatment are critical for the prognosis of cancers [3]. Recently, great progress of diagnosis and treatment in various cancers has been made, however, the prognosis of a large number of individuals remains disappointing [4–7]. Besides, many researchers are focusing on the new biomarkers to elevate the efficacy of diagnosis, prognosis, and treatment of cancers [8, 9]. Nevertheless, the exact mechanism of biomarkers in carcinogenesis is still unclear [10]. Therefore, more and more attention was paid to identify specific biomarkers for prognosis of patients with cancers [11–13].

With the development of technology, long non-coding RNAs (lncRNAs) attract more and more attention of cancer researchers [14–17]. LncRNAs were more than 200 nucleotides in length without the protein-coding function [17]. Recently, the critical roles of some lncRNAs have been confirmed in some tumors, such as gastric cancer [18], lung cancer [19, 20], ovarian cancer [21], liver cancer [22] and so on. However, the detailed information of the most lncRNAs in cancers remains unclear.

LncRNA taurine upregulated gene 1 (lncRNA TUG1) was initially detected in mouse retinal cells, and the expression level could be unregulated with addition of taurine [23]. Recently, a great number of studies indicate that lncRNA TUG1 might participate in progression of a variety of cancers, including non-small cell lung cancer [24], colorectal cancer [25], esophageal squamous cell carcinoma [26], gastric cancer [18] and hepatocellular carcinoma [27] as well as osteosarcoma [28]. Thus to date, the true function of lncRNA TUG1 in cancers was controversial. *Iliev et al.* reported that overexpression of lncRNA TUG1 might predict poor prognosis in high-grade muscle-invasive bladder cancer [29], similar results were reported by *Wang et al.* in clear cell renal cell carcinoma [30]. On the contrary, *Zhang et al.* declared that the lower expression of lncRNA TUG1 was related to higher TNM stage, tumor size and poorer overall survival [18]. In light and consideration of these controversial results, this meta-analysis was performed to explore the prognostic and clinical-pathological significance of lncRNA TUG1 in various cancers.

## MATERIALS AND METHODS

### Literature search strategy

PubMed, Embase and the Cochrane Library database were comprehensively searched up to May 24, 2017. The search strategy was “(((long non-coding RNA) OR lncRNA)) AND ((taurine-upregulated gene 1) OR TUG1)”. All the retrieved papers and their reference lists were carefully checked. The obviously irrelevant articles were directly excluded by scanning the titles or abstracts. The remaining papers were then reviewed comprehensively by carefully reading the full text.

### Inclusion criteria

The studies meeting all the following criteria would be included: 1) prospective or retrospective studies; 2) focusing on the role of lncRNA TUG1 expression on the prognosis in all cancers; 3) reporting enough data to get the hazard ratio (HR) for prognostic outcomes, along with their 95% confidence intervals (CIs) or *P* values; 4) studies published in English.

### Exclusion criteria

The exclusion criteria were as follows: 1) letters, reviews, case reports and expert opinions; 2) without enough data to obtain the HR; 3) not focusing on the prognostic role of lncRNA TUG1 expression on the prognosis in various cancers; 4) not published in English; 5) duplicate publications; 6) reporting the overlapping data.

### Data extraction

All manuscripts were independently reviewed by two investigators. For each included studies, the following data was carefully abstracted: first name of the first author, year of publication, country of the study, ethnicity, the number of patients, percentage of males, tumor type, survival outcomes and analysis model. The HRs of prognostic outcomes obtained directly or indirectly from published articles were integrated in the meta-analysis according to the study conducted by *Tierney et al.* [31]. The HR assessed with multivariate analysis was abstracted when the multivariate analysis and univariate analysis were both applied in the study. The Newcastle-Ottawa Scale (NOS) was used to assess the quality of included studies. And the study with NOS score ≥ 6 was considered to be of high quality. Any other disputes were discussed with the third investigator.

### Statistical analysis

Meta-analysis was carried out by Review Manager Version 5.3 software. The prognosis outcomes were assessed using the HR, along with the corresponding 95% CI or *P* values. The prognosis outcomes mainly contained the overall survival (OS) and some clinical parameters. The Cochran’s *Q* test and Higgins I^2^ were used to assess the heterogeneity among included studies. The heterogeneity should be considered if I^2^ > 50%, and the random-effect model was applied; if not, the fixed-effect model was applied. In additions, the funnel plot was conducted to evaluate publication bias by Review Manager Version 5.3 software. The difference was considered to be significant when *P* value less than 0.05.

## RESULTS

### Literature search

**Figure 1:**
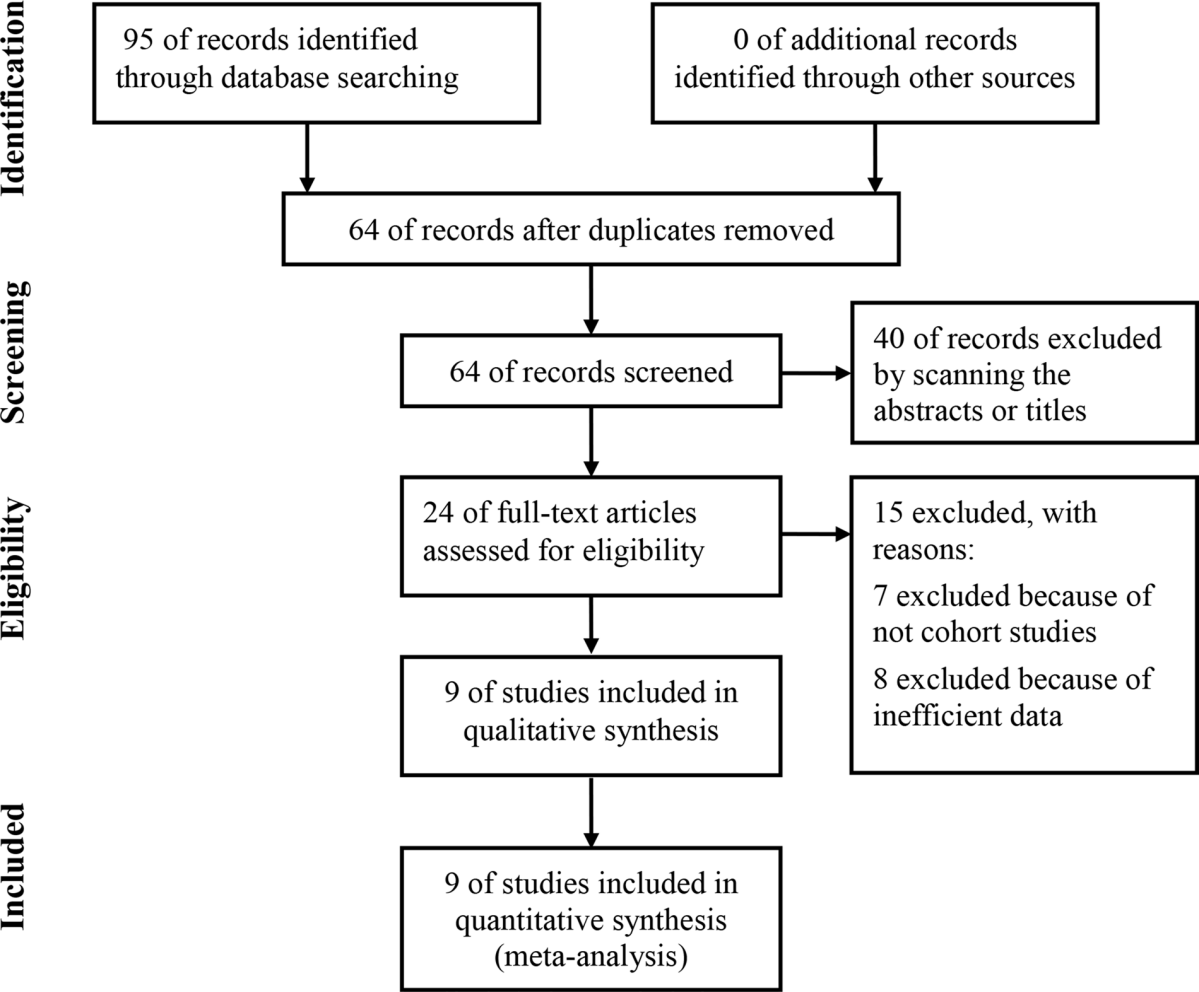
Flow diagram of study selection process

The meta-analysis was conducted according to PRISMA statement (Supplementary Table 1). As shown in Figure 1, 95 articles were initially identified from the PubMed, Embase and Cochrane library. Among the identified articles, 31 articles were excluded for duplications. For the rest articles, 40 articles were excluded by scanning the abstracts or titles. Regarding the rest articles, all of them were analyzed by carefully reading the full text. 7 articles were abandoned because the style of them was not cohort study but also letter, case report or review. Besides, 8 articles were excluded for not reporting the detailed information of the HR of OS. At last, 9 studies involving 1,078 were finally included into the current meta-analysis [18, 24–26, 28–30, 32, 33].

### Characteristics of included studies

The details of the included studies were presented in Table 1. A total of 9 studies involving 1,078 patients were analyzed in the meta-analysis [18, 24–26, 28–30, 32, 33]. Regarding cancer type, the included studies focused on the non-small cell lung cancer (NSCLC) [24, 32], small cell lung cancer (SCLC) [33], bladder cancer [29], gastric cancer [18], esophageal squamous cell carcinoma (ESCC) [26], osteosarcoma [28] and colorectal cancer [25] as well as clear cell renal cell carcinoma (CCRCC) [30]. As for the sample size, the number of included patients varied a lot, from 33 to 218. In additions, 7 studies reported the tumor stage of the included patients [18, 24, 26, 28, 30, 32, 33] and 8 studies reported the detailed follow up time [18, 24–26, 28–30, 32]. As for the therapy, patients enrolled in 5 studies received surgery [18, 25, 28–30] and in 1 study received chemotherapy [26], however, 3 studies didn’t report the detailed information of the therapies [24, 32, 33]. All the included studies use qRT-PCR to detect the expression of LncRNA TUG1 [18, 24–26, 28–30, 32, 33]. As for the clinical outcomes reported in studies, all the included studies reported the OS [18, 24–26, 28–30, 32, 33], however, only 1 study presented the PFS [28]. In additions, OS in 5 studies were assessed with multivariate analysis [18, 24, 26, 28, 30] and 4 studies were assessed with univariate analysis [25, 29, 32, 33]. Regarding quality of included studies, the NOS of all the included studies were more than or equal to 6, which indicated that all the included studies had relatively high quality [18, 24–26, 28–30, 32, 33].

**Table 1:** The main information of included studies in the meta-analysis

Study	Year	Cancer type	Total number	Tumor stage	Follow-up	Therapy	Detection	Clinical	Analysis	NOS
				I–II/III–IV	(months)		method	outcomes		
Zhang et al. [24]	2014	NSCLC	192	129/63	> 60	NA	qRT-PCR	OS,	M	6
Iliev et al. [29]	2016	Bladder cancer	47	NA	42 ± 5	Surgery	qRT-PCR	OS	U	7
Jiang et al. [26]	2016	ESCC	218	96/114	17 (12–72)	Chemotherapy	qRT-PCR	OS	M	7
Lin et al. [32]	2016	NSCLC	89	69/20	> 80	NA	qRT-PCR	OS	U	6
Ma et al. [28]	2016	Osteosarcoma	76	64/12	44 (3–60)	Surgery	qRT-PCR	OS,PFS	M	7
Niu et al. [33]	2017	SCLC	33	16/17	NA	NA	qRT-PCR	OS	U	7
Sun et al. [25]	2016	Colorectal cancer	120	NA	36 (2–60)	Surgery	qRT-PCR	OS	U	7
Wang et al. [30]	2017	CCRCC	203	119/84	> 70	Surgery	qRT-PCR	OS	M	6
Zhang et al. [18]	2016	Gastric cancer	100	57/43	> 60	Surgery	qRT-PCR	OS	M	7

OS = overall survival; NSCLC = non-small cell lung cancer; ESCC = esophageal squamous cell carcinoma; SCLC = small cell lung cancer; CCRCC = clear cell renal cell carcinoma; NA = not available; Multivariate: multivariate analysis; Univariate: univariate analysis; PFS = progression free survival; NOS = Newcastle-Ottawa Scale.

### Association between the lncRNA TUG1 expression and OS

**Figure 2:**
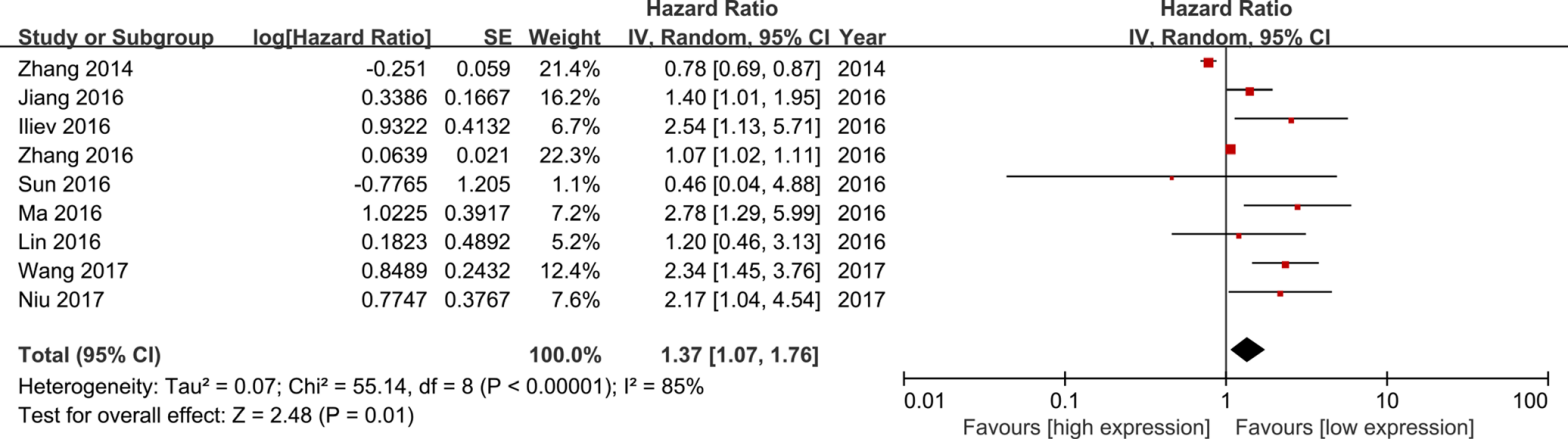
Meta-analysis of overall survival

**Figure 3:**
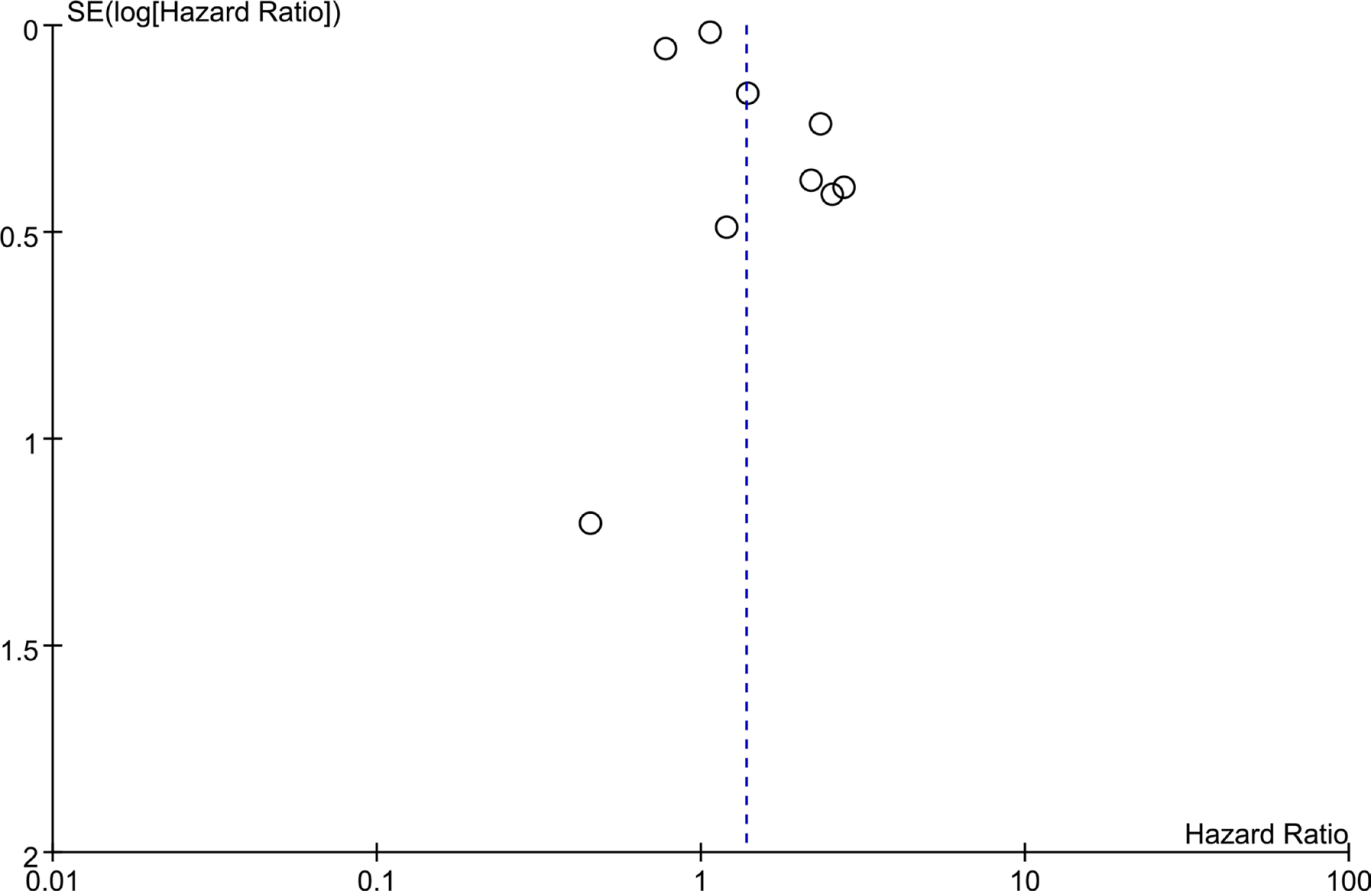
Funnel plot of overall survival

All the included studies reported the information of OS. As shown in Figure 2, the random-effect model was used for the significant heterogeneity (I^2^ = 85%). There was an obvious relationship between the lncRNA TUG1 expression and the OS, and the results presented that patients with low lncRNA TUG1 expression have longer OS when compared to the patients with high lncRNA TUG1 (HR = 1.37, 95% CI = 1.07–1.76, *P =* 0.01).The details of the funnel plot were presented in Figure 3.

### Association between lncRNA TUG1 expression and clinicopathological parameters

**Figure 4:**
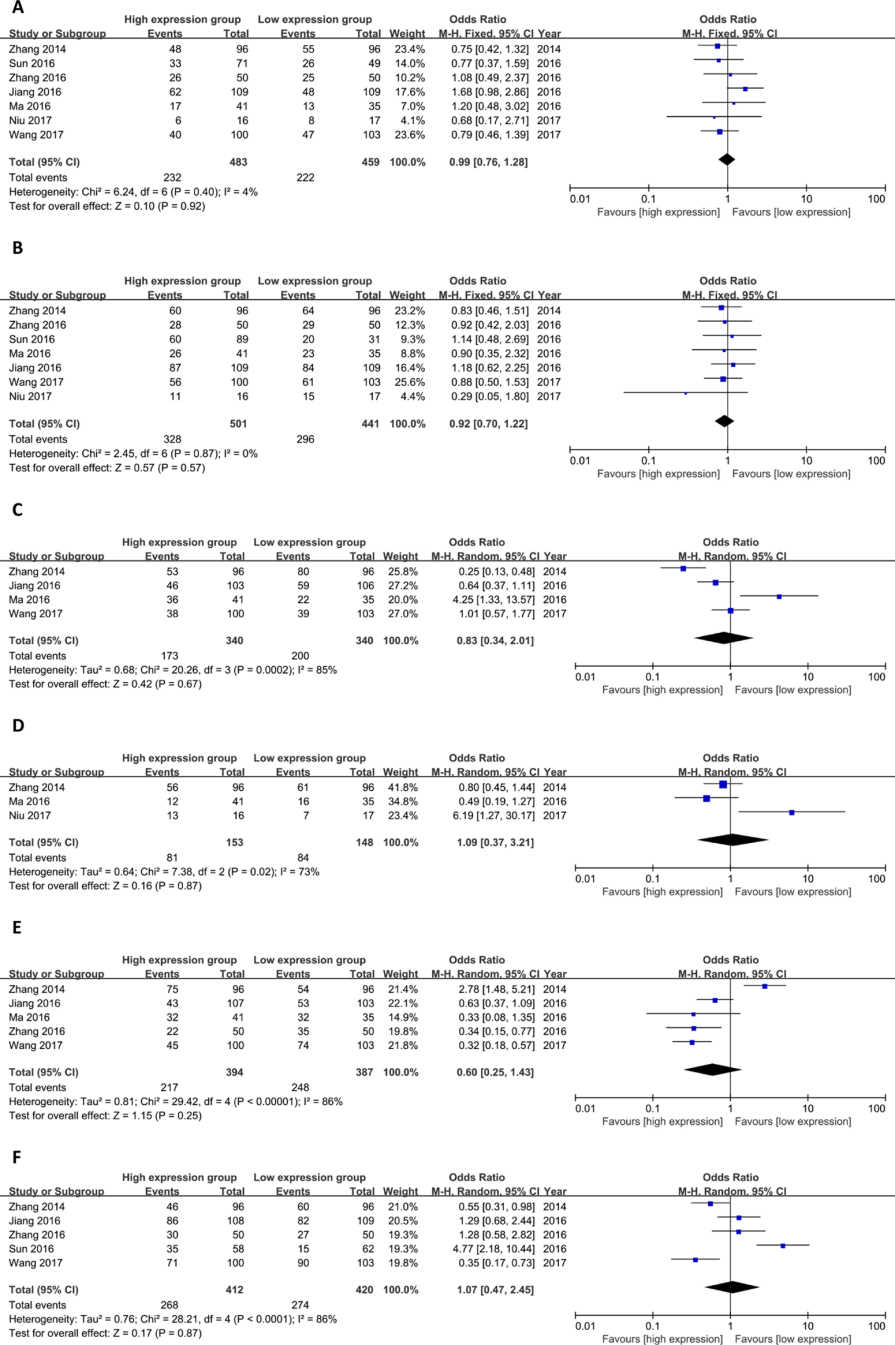
Meta-analysis of clinicopathological parameters (**A** age; **B** gender; **C** diameter; **D** smoking; **E** TNM stage; **F** lymph node metastasis).

**Figure 5:**
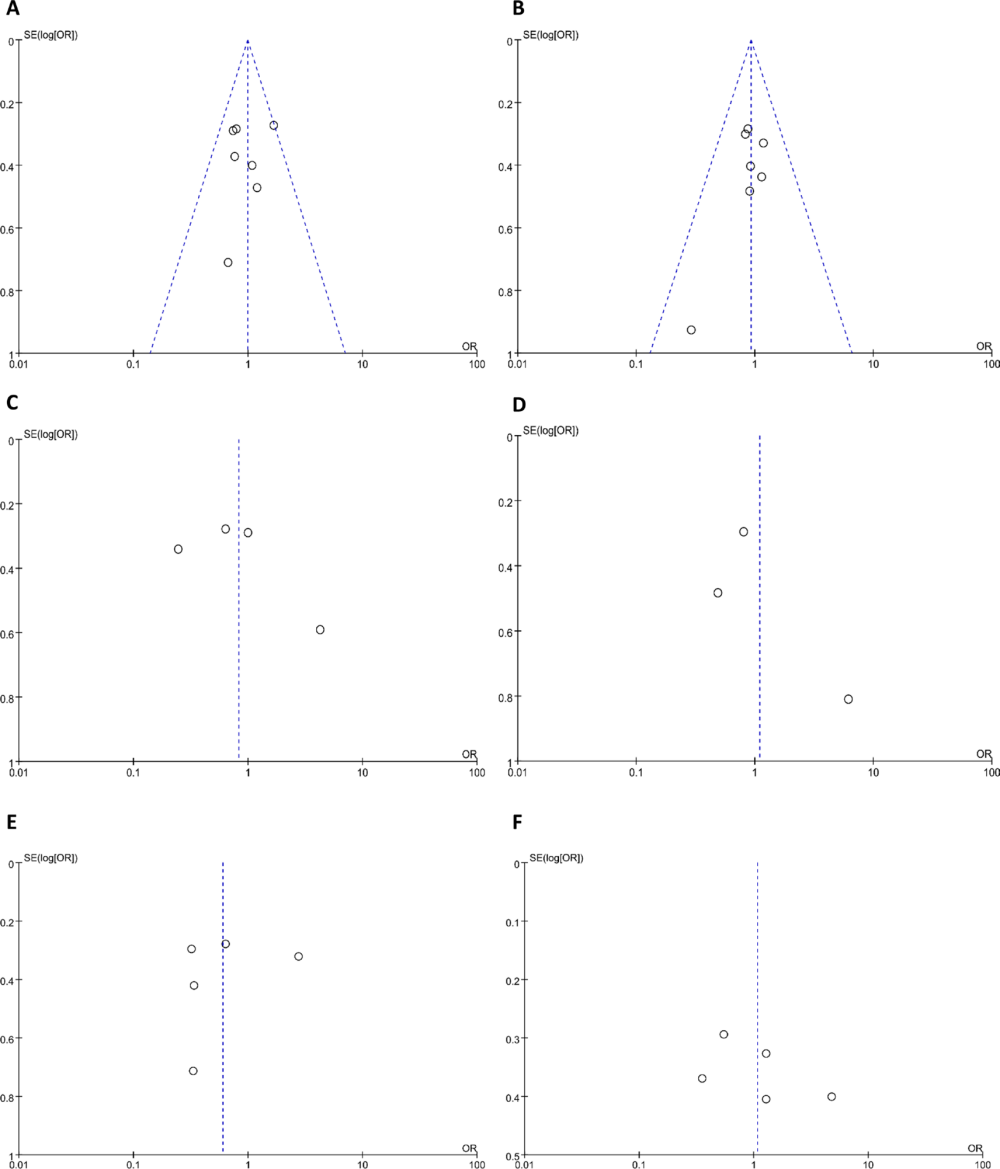
Funnel plot of clinicopathological parameters (**A** age; **B** gender; **C** diameter; **D** smoking; **E** TNM stage; **F** lymph node metastasis).

As shown in Figure 4, the meta-analysis was conducted to explore the association between lncRNA TUG1 expression and clinicopathological parameters. No distinct relationship was observed between the lncRNA TUG1 expression and age (OR = 0.99, 95% CI = 0.76–1.28, *P =* 0.92; I^2^ = 4%), gender (OR = 0.92, 95% CI = 0.70–1.22, *P =* 0.57; I^2^ = 0%), diameter (OR = 0.83, 95% CI = 0.34–2.01, *P =* 0.67; I^2^ = 85%), smoking (OR = 1.09, 95% CI = 0.37–3.21, *P =* 0.87; I^2^ = 73%), TNM stage (OR = 0.60, 95%CI=0.25–1.43, *P =* 0.25;I^2^=86%) and lymph node metastasis (OR = 1.07, 95% CI = 0.47–2.45, *P =* 0.87; I^2^ = 86%). The details of the funnel plots were presented in Figure 5.

## DISCUSSION

With the development of technology, more and more attention was paid to the non-coding RNA [14, 34, 35]. LncRNAs were a class of endogenous RNAs without the protein-coding function, which range from 200 nucleotides to ∼100 kilobases (kb) [36, 37]. Recently, increasing evidences have presented that lncRNAs were involved in the occurrence and progress of several disease, including cancers [37] ,liver disease [38], Parkinson’s disease [39], and especially various cancers [18, 24, 25, 28, 30, 33, 40]. The lncRNAs might promote or prevent the tumorigenesis and development as oncogenes or tumor suppressor, respectively [33, 41].

LncRNA TUG1 was initially observed in mouse retinal cells with the addition of taurine. Increasing evidences have presented that LncRNA TUG1 might play an important role in the tumorigenesis and development of cancers. However, the potential mechanism still remains unclear. *Ma et al.* declared that long non-coding RNA TUG1 might promote cell proliferation and metastasis by negatively regulating miR-300 [42]. LncRNA TUG1 also might be involved in the regulation the expression of miR-335-5p in osteosarcoma cells [43]. Similarly, *Cai et al.* reported that lncRNA TUG1 regulated blood-tumor barrier permeability by targeting miR-144 [44]. Overexpression of lncRNA TUG1 promoted cervical cancer cell proliferation and migration via the progression of epithelial-mesenchymal transition [45]. Meanwhile, *Niu et al.* declared that lncRNA TUG1 was involved in cell growth and chemoresistance of small cell lung cancer by regulating LIMK2b via EZH2 [33]. In ovarian cancer, *Kuang et al.* discovered that lncRNA TUG1 might regulate cancer proliferation and metastasis via altering epithelial-mesenchymal transition [41]. Moreover, *Liu et al.* declared that lncRNA TUG1 could induce apoptosis by targeting ZEB2 mediated by miR-142 through the inactivation of Wnt/β-catenin pathway in bladder cancer [46].

In recent years, numerous studies discovered that LncRNA TUG1 expression was positively associated with prognostic outcomes in cancer patients, however, the results was controversial [29, 30, 32, 33].

In the current study, the results presented that high lncRNA TUG1 expression was an unfavorable prognostic factor in patients with cancers. Besides, no correlation between the lncRNA TUG1 expression and other clinicopathological parameters was observed, including age, gender, smoking, TNM stage, lymph node metastasis and diameter. Regarding PFS, only one study reported the relationship between the PFS and lncRNA TUG1 expression, and the results also indicated that high lncRNA TUG1 expression was an unfavorable prognostic factor. In conclusion, lncRNA TUG1 expression might play an important role in the prognosis of cancers and high lncRNA TUG1 expression might be an unfavorable prognostic factor. To the best of our knowledge, this study was the first meta-analysis to explore the relationship between the lncRNA TUG1 expression and prognosis of patients in cancers. And the results could encourage more researchers pay attention to the prognostic role of lncRNA TUG1 in cancers and further to explore the underlying mechanisms.

Some limitations should be considered of the current study. Firstly, the sample size of all the included studies was relatively small, which might reduce the reliability of the conclusion. Secondly, moderate heterogeneity was observed in some analyses including OS, TNM stage, smoking, and so on, which might influence the convincing of the results. Thirdly, this meta-analysis was only performed to explore the association between the lncRNA TUG1 expression and prognosis of patients in various cancers. No further analysis concerning the association between the lncRNA TUG1 expression and specific cancer was conducted for the limited included studies.

In conclusion, high lncRNA TUG1 expression is an unfavorable predictor of OS in various cancers, and lncRNA TUG1 expression is a promising prognostic biomarker for cancers.

## SUPPLEMENTARY MATERIALS TABLE




