# Supplementary material for: Prognostic role of long non-coding RNA TUG1 expression in various cancers: a meta-analysis
Source: Oncotarget. 2017 Aug 8;8(59):100499–507. doi: 10.18632/oncotarget.20037 (PMC5725038; doi:10.18632/oncotarget.20037)
Supplement: Supplementary file 1 [file oncotarget-08-100499-s001.pdf]

Prognostic role of long non-coding RNA TUG1 expression in various cancers: a meta-analysis

SUPPLEMENTARY MATERIALS

Supplementary Table 1: PRISMA Checklist. See Supplementary_Table_1
